# Effects of seasonal, ontogenetic, and genetic factors on lifespan of male and female progeny of *Arvicola amphibius*

**DOI:** 10.3389/fgene.2013.00100

**Published:** 2013-06-20

**Authors:** G. G. Nazarova

**Affiliations:** Institute of Systematics and Ecology of Animals, Siberian Branch of Russian Academy of SciencesNovosibirsk, Russia

**Keywords:** lifespan, heritability, maternal environment, age of sexual maturity, seasonal cohorts, sex

## Abstract

The water vole (*Arvicola amphibius*) in the forest-steppe of West Siberia is known to have wide fluctuations in abundance. These fluctuations are accompanied by changes in birth and death rates, sex-age structure of the population, and individual morphophysiological and behavioral characteristics of the animals. Survival of the animals depends on season, phase of population cycle, and sex. Based on the data of long-term captive breeding of water voles, the maximal lifespan of males was found to be 1188 days and that of females, 1108 days. There were no differences between the sexes in mean lifespan. The probability of living 2 years or longer was 0.21. Individuals who began breeding at an older age had a significantly longer lifespan and produced more offspring. The survival curves of the spring-born animals were steeper than of those summer-/autumn-born. Maternal factors had a differential effect on males and females with respect to lifespan. Male lifespan correlated negatively with maternal age, parity, and litter size, whereas female lifespan did not correlate with these characteristics. To estimate heritability, parent-offspring correlations of lifespan were calculated, as well as full-sib intraclass correlations. No statistically significant correlation was found for lifespan between sons and mothers, sons and fathers, and daughters and fathers. Daughters' lifespan correlated positively with maternal lifespan (*r* = 0.21, *p* < 0.001). Female full-sibs and male full-sibs had the same intraclass correlations, 0.22, *p* < 0.001.

## Introduction

Population cycles in voles and lemmings are found mainly in northern latitudes (Hansson and Henttonen, 1985; Norrdahl, 1995). In spite of long history studies of this phenomenon, demographic mechanisms of population cyclicity remain insufficiently understood. It is assumed that there are “extrinsic” causes of cyclic fluctuations in animal numbers (Elton and Nicholson, 1942; Erlinge et al., 1983; Sinclair et al., 1993; Krebs et al., 1995; Potapov et al., 2004), as well as “intrinsic” causes, which may act synergistically (Sinclair et al., 2003). The latter are related to behavioral or physiological traits of the animals that can be passed through generations either by genotypic or by maternal processes (Chitty, 1960, 1967; Inchausti and Ginzburg, 2009).

Maternal effects mediated by the age, hormonal, or nutritional state of mothers are considered to be of great significance in inducing a delayed density-dependent feedback on population growth rate (Bernardo, 1996; Rossiter, 1996; Inchausti and Ginzburg, 2009). According to the results of several long-term population studies, mothers from decline phases have lower nutritional conditions, fecundity and quality offspring than those from increase or peak phase of the population cycle (Norrdahl and Korpimäki, 2002; Evsikov et al., 2008; Nazarova and Evsikov, 2012a).

Life history traits, especially age at first reproduction and longevity have a major impact on population growth rate (Krebs and Myers, 1974; Evsikov et al., 1997; Oli and Dobson, 1999, 2001; Erlinge et al., 2000). There are composite, quantitative, polygenic traits whose expression is highly contingent upon plasticity, pleiotropy, and epistasis (Braendle et al., 2011). In rodents, life history traits are characterized by high flexibility and show tremendous temporal and spatial variation (Stearns, 2000; Millar and McAdam, 2001). To understand the peculiarities of self-regulatory mechanisms in population dynamics, it is important to evaluate the heritability of life history traits as well as their dependence on seasonal and maternal environment.

Age at first reproduction and longevity are especially sensitive to factors of population density or seasonal environment (Tkadlec and Zejda, 1998; Erlinge et al., 2000). Tkadlec and Zejda suggested that seasonal environmental variation is from causal factors of bimodality of age at first reproduction and, as a consequence, population cyclicity (Tkadlec and Zejda, 1998). The results of population studies have demonstrated that animals belonging to spring or summer-autumn cohorts differ in growth trajectory, age at first reproduction, hormonal status and rate of ageing (Shvarts et al., 1964; Zejda, 1971; Millar, 1980; Malzahn, 1985; Shintaku et al., 2010). Individuals born in spring months breed in the current reproductive season, whereas those born in summer or autumn months delay maturation until the next year. The proportion of matured young individuals has been found to closely correlate with cyclic fluctuations in abundance (Gliwicz, 1996; Evsikov et al., 1997, 1999; Erlinge et al., 2000).

Some authors have found that in decline phases the average age of the wintering population shift toward older animals due to delayed sexual maturation of young animals and shortening of the breeding season (Zejda, 1967; Wiger, 1979). Boonstra assumed the cause of the declines to be senescence and associated deterioration of physiological functions and fecundity (Boonstra, 1994). Indeed, females trapped during a decline phase and kept in the benign laboratory environment, show poorer reproductive performance, growth, and survival than those trapped during other phases of the population cycle (Mihok and Boonstra, 1992; Boonstra et al., 1998; Nazarova and Evsikov, 2010). However, age-related pattern of reproductive performance in rodents is still poorly understood.

The water vole (*Arvicola amphibius*) provides a good model for the study of internal and external causes of variation of life history traits and their interrelationships. The water vole in the forest-steppe of West Siberia is known to have large-scale fluctuations in abundance. These fluctuations are accompanied by changes in birth and death rates, sex-age structure of the population, individuals' physiological state, and reproductive and behavioral characteristics of the animals (Evsikov et al., 1997; Rogov et al., 1999). As in other cyclic species (Krebs and Myers, 1974), length of the breeding season and the age at sexual maturity change markedly during the population cycle. In decline, breeding season is 2 months shorter than in the increase phase, and therefore, overwintering females make a major contribution to reproduction. Due to extremely low reproductive output (about 1 young caught per 10 reproductively active females) (Rogov, 1999), population existence after population crash is highly dependent on the persistence of individuals capable of surviving adverse conditions of a decline phase.

In wild populations, the survival of water voles depends on season, phase of population cycle, and sex of animals (Rogov et al., 1999). In captivity, seasonal patterns of growth, maturation and reproductive activity are similar to that observed in the wild, providing an opportunity to evaluate the impact of season of birth and several characteristics of maternal environment on variability of life history traits.

Longevity is a critical parameter of fitness, determining population growth rates, abundance, and sex structure of the population. Because studies dealing with variability of lifespan in myomorphic rodents associated with individual morphophysiological and genetic traits and sex are scare, this long-term multigenerational study conducted on captive-bred water voles aimed (1) to clarify the effects of season of birth, age of sexual maturity, and some characteristics of family environment on lifespan and (2) to evaluate heritability of lifespan. The results help understand the internal mechanisms underlying the dynamics of a cyclic rodent population and evolution of lifespan.

## Materials and methods

The study was conducted on an outbred colony of water voles (*Arvicola amphibius*), established in 1984 in the vivarium of the Institute of Systematics and Ecology of Animals. Their ancestors were taken from the cyclic population near Lis'yi Norki village (55° 50′N, 80° 00′E), Novosibirsk oblast. Each 1 to 3 years, new animals from the source population were added to the established colony to limit inbreeding.

The animals were kept in separate 48 by 25 by 25 cm hay-bedded cages under natural photoperiod (55° 1′N, 82° 55′E), with *ad*-*libitum* access to food (stewed grains, carrots, and cereal germs) and water.

During the breeding season (March–October), females and males were paired, for which their separate cages were connected with 8 by 6 cm tubular passages which were removed 10–18 days after mating. To verify copulation by the presence of sperm, vaginal smears were taken daily for 2 weeks of pairing. The young were weaned when they were 20 or 21 days old and were placed in separate cages. Females unmated for two weeks were caged with a different male. During breeding season, each individual was paired with 2–3 mates on average. Coefficient of relationship of mates was not greater than 0.125.

Survival and lifespan were estimated only in ≥20-day-old animals. Mean, median, and maximal lifespan were estimated from life table data composed of a total of 2016 observations (1424 uncensored and 592 censored).

The data on 1013 males (74.3% of the uncensored observations) and 1003 females (66.9% of the uncensored observations) were used for survival analyses of spring- and summer-/autumn-born individuals. The former were the individuals born in March–May (386 males and 380 females). The latter were those born in June–October (627 males and 623 females). Survival curves were constructed using the Kaplan-Meier method, and intergroup differences were evaluated using Cox's *F*-test.

The narrow-sense heritability of lifespan was estimated by doing lifespan regressions of an average offspring on each parent or by calculating intraclass correlations of lifespans of full sibs (for males and females separately) and multiplying the values obtained by 2 (Falconer, 1989). Only uncensored data were used for this purpose. A total of 355 mother–son and 365 father–son pairs, 354 mother–daughter and 335 father–daughter pairs, 202 sibs male groups (2 to 6 individuals each), and 166 female sibs groups (2 to 5 individuals each) were studied. Standard errors of the intraclass correlation were determined using the formula in Swiger et al. (1965).

The data were analyzed using one-way analysis of variance, survival analysis, and Spearman rank correlations. In the text and tables, means were given with their standard errors. Probabilities of less than 0.05 were accepted as significant. Statistical differences between the means were estimated by the Mann–Whitney *U*-test and Student *t*-test. The statistical package Statistica 6.0 was used for all computations.

## Results

### Mean and maximal lifespan of males and females

The analysis of life tables showed that the median lifespan was 421.8 days, with the 25th and 75th percentiles being 273.0 and 679.0 days, respectively. The probability of living 2 years or longer was 0.21 ± 0.01.

**Figure 1:**
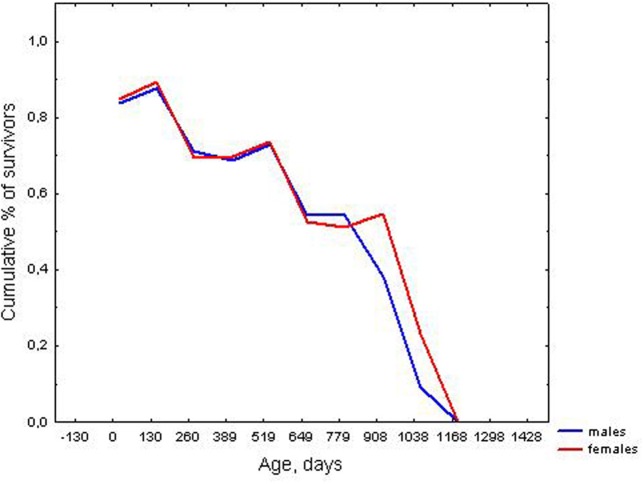
**Survival curves for males and females**.

Males and females showed no statistically significant differences in mean lifespan calculated for uncensored data: 393.2 ± 9.6, *n* = 753 and 367.6 ± 9.1, *n* = 671, respectively (*U* = 241742, *z* = 1.40, *p* > 0.05), and had a similar median and maximal lifespan (Table 1). However, according to the Cox test, a difference in survival curves was found between males and females, [*T*_1_ = 699.8, *T*_2_ = 725.2, *F*_(1342, 1506)_ = 1.16, *p* = 0.002], especially in older ages. Kaplan–Meier survival curves for males and females are shown in Figure 1.

**Table 1:** **Mean, median, and maximum life span**.

**Sex**	**No. of individuals**	**25%**	**50%**	**75%**	**Maximum**
Males	1013	264.9	419.5	681.2	1188
Females	1003	286.5	423.8	678.7	1108

### Reproductive characteristics of 1- and 2-year-old individuals

Female reproductive capacity did not deteriorate with age. One- and two-year-old females did not significantly differ in the percentage of mated females that delivered litters and average litter size at birth. Two-year-old males had lower percentage of sires than one-year-old ones (Table 2).

**Table 2:** **Reproductive characteristics of females and males aged one and two years**.

**Sex**	**Females**		**Males**	
Age, years	1	2	1	2
No. of animals	63	63	68	68
No. of mated animals (% ± *SE*)	49 (77.8 ± 5.2)	55 (87.3 ± 4.2)	58 (85.3 ± 4.3)	50 (73.5 ± 5.4)
No. of sires or dams (% ± *SE*)	42 (66.7 ± 5.9)	41 (65.1 ± 6.0)	51 (75.0 ± 5.2)^*^	36 (52.9 ± 6.0)
Total no. of litters	79	70	95	50
Litter size at birth	4.4 ± 0.2	4.2 ± 0.2	4.3 ± 0.2	4.5 ± 0.3

* p < 0.05.

#### Correlation between lifespan and reproductive characteristics

**Figure 2:**
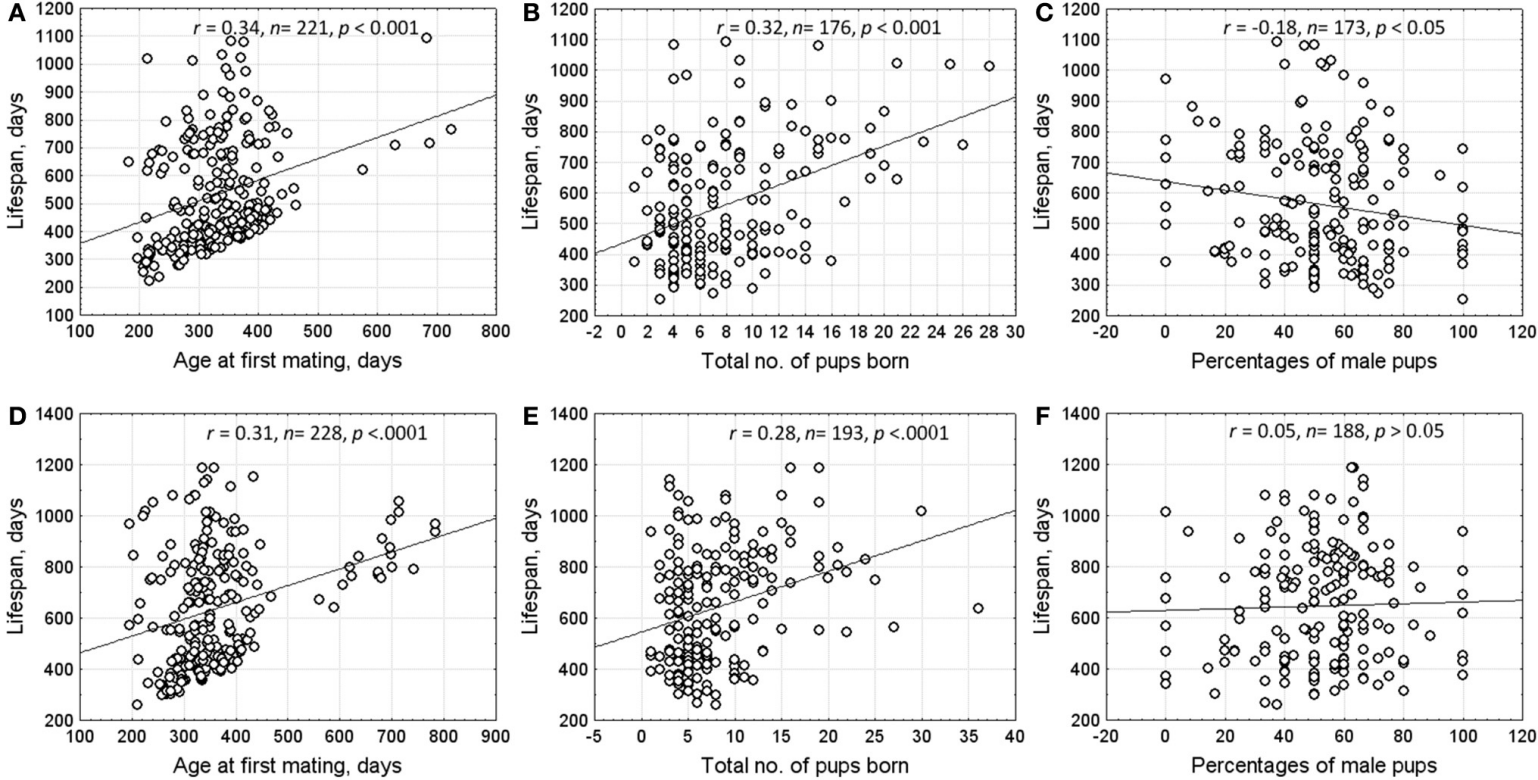
**Correlations between lifespan and reproductive characteristics of males and females**. Upper row—females **(A)** age at first mating; **(B)** total no. of pups born; **(C)** percentage of male pups). Lower row—males **(D)** age at first mating; **(E)** total no. of pups born; **(F)** percentage of male pups.

The correlation between lifespan and reproductive characteristics (age at first mating, number of pups born throughout life, and offspring sex ratio (% male pups) in all litters are shown in Figure 2. Spearman rank correlation was performed on uncensored data only. The results indicated that individuals who began breeding at an older age had a significantly longer lifespan. Lifespan of both males and females correlated positively with the number of pups born throughout life. However, females who had more sons in progeny had significantly shorter lifespan.

#### Effects of maternal environment on lifespan

Male lifespan correlated negatively with maternal age, parity, and litter size. These characteristics accounted for about 1% of variance of male lifespan. Female lifespan did not correlate with these maternal characteristics (Table 3).

**Table 3:** **Spearman rank correlations between lifespan and maternal environment**.

**Sex**	**Maternal age**	**Parity**	**Litter size**
Males	−0.11^***^	−0.14^***^	−0.10^*^
	(672)	(667)	(674)
Females	Ns	Ns	Ns
	(596)	(587)	(596)

* p < 0.05;

*** p < 0.001. Ns, non-significant.

### Survival of spring- and summer/autumn-born individuals

**Figure 3:**
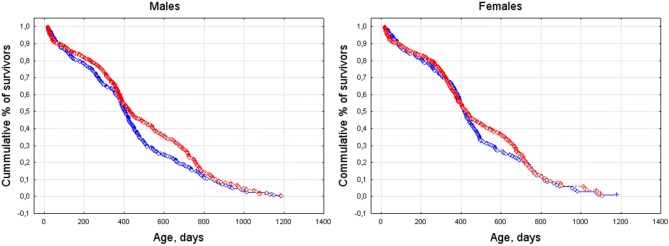
**Survival curves for spring- and summer-/autumn-born males (left) and females (right)**. Blue line: born March–May, red line: born June–October.

According to Cox's *F*-test, males and females showed significant differences between survival curves for spring- and summer-/autumn-born individuals [males—*F*_(846, 660)_ = 1.53, *p* < 0.001; females—*F*_(780, 562)_ = 1.27, *p* = 0.001]. The survival curves of spring-born animals were steeper than of those of summer-/autumn-born (Figure 3).

### Heritability of lifespan

#### Parent–offspring correlation

**Figure 4:**
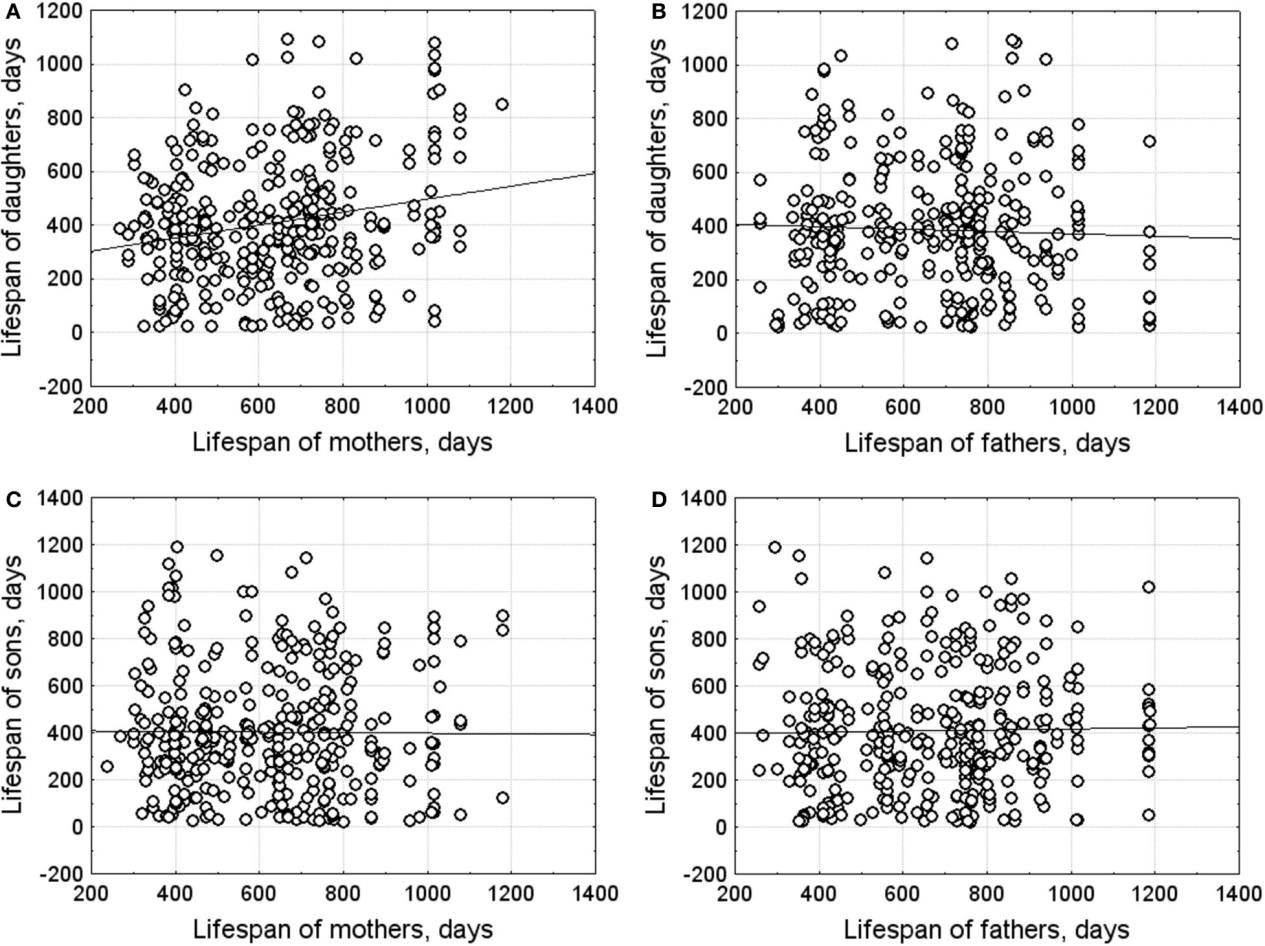
**Parent-offspring correlations of lifespan. (A)** mother-daughter; **(B)** father-daughter; **(C)** mother-son; **(D)** father-son.

Parent-offspring correlations of lifespan are presented in Figure 4. Lifespan of daughters correlated positively with maternal lifespan (*r* = 0.21, *n* = 354, *p* < 0.001) and did not correlate with paternal lifespan (*r* = −0.04, *n* = 335, *p* < 0.455). Heritability, calculated as the mother–average daughter regression multiplied by 2, was 0.46 ± 0.14 (*p* < 0.001).

No significant correlation was found between sons' and parental lifespans (mother–son: *r* = 0.04, *n* = 355; father–son: *r* = 0.02, *n* = 365).

#### Sib lifespan correlations

One-Way ANOVA revealed a significant sibship effect on lifespan of female [*F*_(166, 252)_ = 1.72, *p* < 0.001] and male [*F*_(201, 312)_ = 1.71, *p* < 0.001] progeny. Female and male sibs had the same intraclass correlations, 0.22 ± 0.003. Heritability was 0.44 ± 0.006.

Heritability of lifespan for sons, assessed from full-sib intraclass correlation, significantly exceeded that determined by parent–offspring regression (*p* < 0.001).

## Discussion

Differential survival of males and females is a major factor influencing the sex ratio of adult animals in wild populations of the water vole (Rogov et al., 1999). The main purpose of this study was to examine gender differences in lifespan and their dependence on season of birth and factors of maternal environment.

### Lifespan and survival curves of males and females

In captivity, maximal lifespan of males was 1188 days and that of females, 1108 days. Mean lifespan did not differ between the sexes. The lack of differences in mean lifespan between the sexes has been noted in *Microtus ochrogaster, M. pennsylvanicus*, and *M. townsendii* (Boonstra, 1994; Getz et al., 1997).

However, there was a statistically significant difference in the survival curves of males and females, with males having a lower survival probability in older ages, than females. The observed gender difference in survival curves can be accounted by the more rapid progression of senescence in males than females.

### Age-related reproductive patterns in males and females

Lifespan is an important component of individual fitness. In water voles, 20% of individuals lived longer than 2 years and most of them maintained reproductive ability. As a result, long-lived animals produced more offspring. The observed negative correlation between lifespan of females and the proportion of males in their progeny can be attributed to a higher physiological cost of rearing sons than daughters (Evsikov et al., 1994). Reproductive investment to predominantly male progeny shortens the lifespan of mothers.

The results of analysis of age-related variation of reproductive characteristics indicated that there was no evidence of reproductive senescence in females. One- and two-year-old females did not significantly differ in the percentage of mated females that delivered litters and average litter size at birth. Similar results were obtained in Richardson's ground squirrel and Siberian lemming females (Erlinge et al., 2000; Broussard et al., 2005). However, in deer mice and white-footed mice (Millar, 1994; Morris, 1996), older females have decreased reproductive success.

As for water vole males, their reproductive capacity decreased with advancing age. It is a common feature of most polygynous vertebrates (Clutton-Brock and Isvaran, 2007).

### Effect of season of birth on lifespan

Arvicoline rodents in high-latitude environments are known to undergo pronounced seasonal changes in their physiology (Bronson, 1985; Ebling and Barrett, 2008). Spring- and summer-/autumn-born individuals are known to exhibit considerable biological differences, as in natural populations, as in captivity (Shvarts et al., 1964; Panteleev, 1968; Getz et al., 1997).

In this study, statistically significant differences in survival curves between the spring-born and summer-/autumn-born cohorts were observed in males and females. The curves of spring-born individuals showed a steeper decline than those of summer-/autumn-born ones. This appears to be determined by physiological response of an organism to yearly changes of day length, because other environmental conditions were under control.

The potential role of day length in the regulation of lifespan was experimentally shown in mice by Blom et al. (1994). The authors found that the immune status of offspring is affected by prenatal photoperiod. It is lower in mice carried under long prenatal photoperiod than in those carried under a short one. Other studies in *Microtus montanus* and *Microtus pennsylvanicus* showed that some characteristics of life history, correlating with lifespan, such as growth and sexual maturation, are also influenced by the prenatal and postnatal photoperiod (Horton, 1984; Lee et al., 1987).

### Age at first reproduction and lifespan

Costs and benefits of early and delayed maturation play an important role in the evolution of life history. The cost of reproduction usually increases with decreasing age at first reproduction (Adams, 1985). The extensive comparative studies conducted in mammals support that species-specific lifespan is inversely related with age of sexual maturity and fecundity (Severtsov, 1930; Clutton-Brock and Isvaran, 2007; De Magalhães et al., 2007; Jones et al., 2008). Kirkwood (1977) hypothesized that lifespan correlated negatively with ratio of expenditures devoted to growth and reproduction on the one hand and maintenance of body integrity and organism viability on the other hand. The results obtained in this study revealed that early reproduction impaired organism viability and shortened lifespan. A similar phenomenon was found for females from the cyclic population of water voles: the younger the age of a female at first reproduction, the fewer such females survived winter (Rogov, 1999).

### Effects of maternal characteristics on lifespan

The analysis of the effects of maternal environment on lifespan revealed a weak negative correlation between male lifespan and maternal age, parity, and litter size. Female lifespan did not correlate with these characteristics. Therefore, maternal environment has a different effect on males and females in terms of survival and lifespan. However, several recent studies on mice have demonstrated an opposite tendency: lifespan of females is more affected by maternal age (Carnes et al., 2012).

Negative correlation between male lifespan and litter size at birth would imply the existence of a trade-off between offspring number and offspring quality (Smith and Fretwell, 1974).

### Heritability of lifespan

Lifespan heritability, evaluated from the mother–average daughter regression and showing the percentage of additive genetic variance in the total phenotypic variance, was 0.46. Lifespan heritability, estimated from intraclass correlation of full-sib females, was 0.44. These values are similar to those obtained in mice (Klebanov et al., 2000). The correlation between daughters' and paternal lifespan was nearly zero. The difference between “daughter-mother” and “daughter-father” correlation coefficients indicates that female longevity could depend on family environment or inheritable maternal physiological qualities that affect both reproductive success and probability of death associated with reproduction, for example, the ability to accumulate body reserves during pregnancy for lactation needs (Nazarova and Evsikov, 2012b). It is known, that reproduction has considerable energy demands in mammals and is risky for females (Gittleman and Thompson, 1988).

The ecological factors of local environment and individual maternal characteristics play an important role in fulfillment of reproductive potential and demographic dynamics of the water vole (Nazarova and Evsikov, 2000, 2004, 2007, 2008, 2012a; Muzyka et al., 2010). During breeding season, reproductive groups composed of males and females of various ages form the basic unit of the spatial organization of a water vole population (Evsikov et al., 2001; Muzyka et al., 2010). These groups may have different reproductive success and duration. Breeding females have non-overlapping home ranges and produce several litters per breeding season. Before birth, pregnant voles move to a new territory, leaving their older offspring in their natal territory (Waser and Jones, 1983; Jeppson, 1986). Daughters of many rodent species are more likely to remain within the maternal home range than sons (Solomon and Keane, 2007). The present results showed that water vole females inherited not only the home range but also qualities that determine longevity.

Sons' lifespan heritability estimated from the parent–offspring regressions did not differ significantly from zero. Lifespan heritability, calculated from intraclass correlations between full-sib males, was 0.44, the same for males and females. The higher sib-sib than parent-offspring heritability coefficients may be accounted by effects of the common rearing environment.

In conclusion, developmental factors have substantial influence on water vole longevity. Potentially, phase-related changes in characteristics of maternal environment or mean age at first reproduction of females could be primary factors influencing the sexual structure of populations.

### Conflict of interest statement

The author declares that the research was conducted in the absence of any commercial or financial relationships that could be construed as a potential conflict of interest.
